# Terpene Coordinative Chain Transfer Polymerization: Understanding the Process through Kinetic Modeling

**DOI:** 10.3390/polym14122352

**Published:** 2022-06-10

**Authors:** Andrés Ubaldo-Alarcón, Florentino Soriano-Corral, Teresa Córdova, Iván Zapata-González, Ramón Díaz-de-León

**Affiliations:** Centro de Investigación en Química Aplicada, Enrique Reyna Hermosillo, No.140, Col. San José de los Cerritos, Saltillo 25294, Mexico; aubaldo.d18@ciqa.edu.mx (A.U.-A.); florentino.soriano@ciqa.edu.mx (F.S.-C.); teresa.cordova.ps@ciqa.edu.mx (T.C.)

**Keywords:** myrcene, CCTP, kinetic modeling

## Abstract

The interest in the Coordinative Chain Transfer Polymerization (CCTP) of a family of naturally occurring hydrocarbon monomers, namely terpenes, for the production of high-performance rubbers is increasing year by year. In this work, the synthesis of poly(β-myrcene) via CCTP is introduced, using neodymium versatate (NdV_3_), diisobutylaluminum hydrade (DIBAH) as the catalytic system and dimethyldichlorosilane (Me_2_SiCl_2_) as the activator. A bimodal distribution in the GPC signal reveals the presence of two populations at low conversions, attributable to dormants (arising from reversible chain transfer reactions) and dead chains (arising from termination and irreversible chain transfer reactions); a unimodal distribution is generated at medium and high conversions, corresponding to the dominant species, the dormant chains. Additionally, a mathematical kinetic model was developed based on the Method of Moments to study a set of selected experiments: ([*β-myrcene*]_0_:[*NdV_3_*]_0_:[*DIBAH*]_0_:[*Me_2_SiCl_2_*]_0_ = 660:1:2:1, 885:1:2:1, and 533:1:2:1). In order to estimate the kinetic rate constant of the systems, a minimization of the sum of squared errors (SSE) between the model predicted values and the experimental measurements was carried out, resulting in an excellent fit. A set of the Arrhenius parameters were estimated for the ratio [*β-myrcene*]_0_:[*NdV_3_*]_0_:[*DIBAH*]_0_:[*Me_2_SiCl_2_*]_0_ = 660:1:2:1 in a temperature range between 50 to 70 °C. While the end-group functionality (EGF) was predominantly preserved as the ratio [*β-myrcene*]_0_:[*NdV_3_*]_0_ was decreased, higher catalytic activity was obtained with a high ratio.

## 1. Introduction

The growing interest in rare-earth metal-based catalytic systems for the polymerization of 1,3-conjugated dienes, such as 1,3-butadiene, isoprene and terpenes, to produce high-performance rubbers is due to their high catalytic activity and a high degree of stereoregulation [1,2,3,4,5]. The polymers obtained using these types of catalytic systems are characterized by broad molecular weight distributions, as a result of the formation of catalytic species with different activity, and also because of the irreversibility of chain transfer reactions during the polymerization. However, by altering some polymerization reaction conditions, especially the ratios [*cocatalyst*]/[*catalyst*] and [*halide donor*]/[*catalyst*], it has been possible to change the irreversibility of the chain transfer, giving place to the coordinative chain transfer polymerization (CCTP). This mechanism confers living characteristics on to the polymerization, where that cocatalyst can also act as a chain transfer agent (CTA), exhibiting three main features which define the CCTP and can be associated with characteristics of living polymerizations: (i) the average number molecular weight (*M_n_*) of the polymer must have a linear relationship to the polymer conversion; (ii) the molecular weight distribution (MWD) of the polymer produced must be narrow, usually with dispersities (Ð) less than 2; and (iii) the number of polymer chains produced per single primary metal atom (*Np*) should be between 4 and 10. The CCTP of butadiene and isoprene has been carried out with very promising results [6,7,8,9,10]; nevertheless the potential of this kind of polymerization can be successfully extended to the stereoregulated polymerization of terpenes, such as β-myrcene.

On the other hand, kinetic analyses of CCTP have been extensively carried out for olefins using mathematical modeling as a tool. A kinetic Monte Carlo (KMC) model was developed for the copolymerization of ethylene and 1-octane CCTP in semibatch operation [11,12,13]. The simulation comprises microstructure details, molecular weight distributions and copolymer composition distributions. Recently, a hybrid programming between KMC and artificial neural network modeling has been reported to take advantage of both simulation techniques [14], and the connection between the reagent ratios, the topology and the property-related microstructural features was explored. Deterministic approaches have also been developed to study the CCTP processes. Most of the deterministic models developed to describe this process are based on the Method of Moments [15,16,17], since it is a great and versatile tool widely used by the polymerization reaction engineering community to develop an improved fundamental understanding of complex mechanistic schemes and new polymerizations. The isoprene polymerization under CCTP conditions with the ternary Ziegler–Natta catalyst system composed of neodymium versatate (NdV_3_), diisobutylaluminum hydrade (DIBAH) and dimethyldichlorosilane (Me_2_SiCl_2_) was simulated, the kinetic rate parameters were estimated, and the evolution and the conversion and average molecular weights fitted in good way to the experimental data [16].

In this study, we introduce the β−myrcene polymerization under CCTP conditions, using NdV_3_, DIBAH and Me_2_SiCl_2_ as the catalytic system. A wide range of operating reaction conditions and reagent ratios were explored and the influence on the monomer conversion and the average molecular weights is analyzed. A reaction mechanism is proposed based on the experimental findings and a kinetic model is developed, based on the Method of Moments. The set of the kinetic rate constants are estimated on the basis of optimization to minimization of the sum of squared (relative) errors (SSE) between the model predicted values and the experimental measurements. The model describes the monomer conversion, the average molecular weights for each type of active chain, the average molecular weight of the product, the dispersities, the number of polymer chains per neodymium atom (*Np*), and the end-group functionality (EGF). Then, the effect of the ratio [*M*]_0_/[*Nd*]_0_ and operating temperature is correlated with the average molecular properties, end-group functionality and the values of *Np*.

## 2. Materials and Methods

### 2.1. Reagents and Materials

The β-myrcene monomer (purity of ≈89%, acquired from VENTOS, Barcelona, Spain) was distilled under vacuum pressure in the presence of metallic sodium before use. To remove the organic (aromatic) traces in the industrial-grade cyclohexane, it was washed with concentrated sulfuric acid (200 mL of sulfuric acid per 1 L of cyclohexane) and then cleaned with distilled water until neutral pH was obtained. Afterward, the cyclohexane was dried by double distillation with reflux: the first with lithium aluminum hydride and the second with metallic sodium, all in a nitrogen atmosphere. Both the cyclohexane solvent and distilled β-myrcene were stored in a stainless steel container with a nitrogen atmosphere.

The catalyst system comprised NdV_3_ as the catalyst (0.54 M solution in hexanes, reagent grade, obtained from Solvay, Jalisco, Mexico), DIBAH as the cocatalyst (molarity 0.95–1.10 mol L, acquired from Sigma Aldrich, Burlington, MA, USA) and Me_2_SiCl_2_ (purity > 99.50%, acquired from Sigma Aldrich) as the halide donor. Using distilled cyclohexane, a 0.22 M solution of the Me_2_SiCl_2_ was prepared. Tetrahydrofuran (THF) HPLC grade (purity > 99.90%, acquired from Sigma Aldrich) was used as the eluent and to prepare the GPC samples.

### 2.2. Catalytic System

The preparation of the NdV_3_/DIBAH/Me_2_SiCl_2_ catalytic system was carried out in a glove box under a nitrogen atmosphere. DIBAH was added dropwise to a glass vial, which had been subjected to anhydrous and anaerobic treatment, at a ratio of 20:1 with respect to NdV_3_, and immediately afterward the NdV_3_ was added, which was left stirring for 2 min. Then, Me_2_SiCl_2_ was added in a molar ratio of 1:1 with respect to the NdV_3_. The catalytic system was aged for 30 min with stirring at room temperature, in order to obtain a pre-aged catalyst.

### 2.3. Polymerization System

The polymerization reactions were performed in a stainless steel reactor of 1 L, which was equipped with a turbine-type mechanical agitation system and a heating mantle coupled with a PDI temperature controller. The temperature controller allowed the maintenance of the temperature of the reactor with a variation lower than 1.5 °C with respect to the set point. The reactor is coupled to a system that allows feeding appropriated amounts of solvent and monomer to the reactor under an inert atmosphere.

### 2.4. Polymerizations

Before each polymerization reaction, the reactor was heated to 120 °C, under a three- cycle protocol of nitrogen loading and vacuum, to eliminate oxygen and humidity traces. Polymerizations of β-myrcene were performed at 16.0% by mass of monomer, using cyclohexane as a solvent, and were carried out under nitrogen atmosphere and isothermally, maintaining constant stirring at 80 rpm. Three temperatures were set for performing the polymerizations: 50, 60 and 70 °C.

For carrying out the polymerizations, cyclohexane (310 mL) and β−myrcene (40 mL) were charged to the reactor and it was brought to the reaction temperature. Then, the pre-aged catalytic system (NdV_3_/DIBAH/Me_2_SiCl_2_) was incorporated into the reactor to start the polymerization, and the reactor was pressurized immediately to 30 psi of nitrogen. Once the reaction of β-myrcene had started, it was carried out isothermally.

### 2.5. Determination of Conversion and Molecular Weight

Samples were taken every certain time, depending on the progress of the polymerization, to measure the conversion and the average molecular weight. The polymerization conversion was determined gravimetrically.

Gel Permeation Chromatography (GPC) was used to obtain the number average molecular weight (*M_n_*), weight average molecular weight (*M_w_)* and dispersity (*M_n_*/*M_w_*). GPC was carried out in an Agilent Technologies model PL-GPC 50, equipped with an index detector refraction calibrated with monodispersed polystyrene standards, using THF as the eluent at a flow rate of 1 mL/min at 40 °C. *M_n_* and *M_w_*, respectively, relative to polystyrene standards, were corrected using the Mark–Houwink–Sakurada equation (Equation (1))

(1) $$\left[\eta \right]=KM^{\alpha }$$

where [*η*] is the intrinsic viscosity, *M* is the viscosimetric average molar mass of the polymer, and *K* and *α* are Mark–Houwink–Sakurada constants, and vary with polymer type, solvent and temperature. The correction was based on a universal parameter known in SEC calibration (the product [*η*]*∙M* is proportional to the hydrodynamic volume). At any retention volume, the hydrodynamic volumes of two polymers 1 and 2 will be equal [18]. The *M* of polymer 2 (*M*_2_), which elutes at the same retention volume of polymer 1 in the same solvent and at the same temperature, is related to the *M* of polymer 1 (*M*_1_) according to

(2) M2=K1M1α1+1K21α2+1

The Mark–Houwink–Sakurada parameters used in the correction were obtained from González-Villa et al. [19], and Hattam et al. [20], using THF as the solvent; Polystyrene *α_PS_* = 0.712, *K_PS_* = 12.80 × 10^−5^ dL/g, *α_MY_* = 0.772, *K_MY_* = 7.46 × 10^−5^ dL/g.

### 2.6. ^1^H and ^13^C NMR

The microstructure of the resulted poly(β-myrcene) samples was calculated by ^1^H and ^13^C nuclear magnetic resonance (NMR) acquired in a Bruker BioSpin-400 MHz spectrometer, using 16 and 140,000 scans, respectively. CDCl_3_ was used as a solvent and the analyses were performed at 25 °C. A concentration of 15 mg of sample in 1 mL of CDCl_3_ was used for the ^1^H-NMR spectra and 80 mg of sample in 1 mg of CDCl_3_ for the ^13^C-NMR spectra.

## 3. Mathematical Modeling

### 3.1. Reaction Mechanism

**Figure 1:**
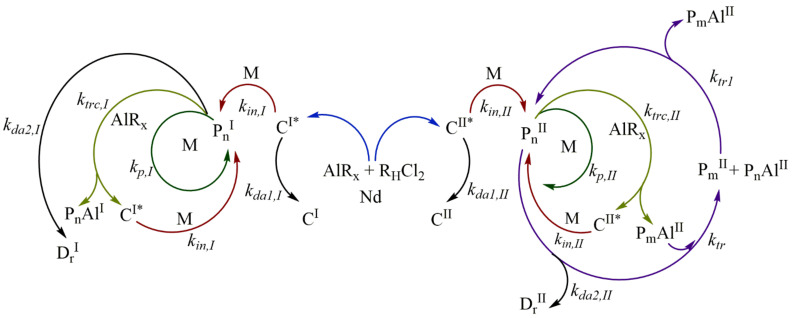
Proposed reaction mechanism for the myrcene polymerization by CCTP.

Polymerization is catalyzed by the neodymium-based Ziegler/Natta catalyst systems via a coordination mechanism, as shown in Figure 1. The catalytic system is comprised of a neodymium catalyst (*Nd*), an alkyl-aluminum species (*AlR_x_*) used as the cocatalyst, which is used also as a chain transfer agent during polymerization, as well as a halide donor (*R_H_Cl_2_*) to achieve high catalytic activities and high *cis*-1,4-contents [21]. The catalyst is firstly activated with the alkyl-aluminum species, which also acts as a scavenger for impurities, and with the halogen donor, to generate the active species or active catalyst sites, which can initiate the polymerization [10,22].

There is evidence for more than one type of active catalytic center in the neodymium-based catalyst systems; many studies have identified traces of bimodal MWD in SEC with these catalyst systems, particularly at low conversion [23,24]. Two different types of active centers are believed to operate in coordination polymerization when neodymium-based catalyst systems are used. It has been proposed that a rapid initial polymerization occurs in insoluble particles (fast but short-lived active centers, C^I*^) which may not be visible to the naked eye. In addition to the initial polymerization, slower rate polymerization occurs at the second type of “soluble” catalyst site (slow-growing but stable active centers, C^II*^) [23,24].

The type I active centers C^I*^ and the type II active center C^II*^ generate several polymer species of Type *I* and Type *II* through different reaction stages, and as the first approach to understand the mechanism, it is assumed that such species only react between the same type and that these can be mechanistically separated.

The initiation step is based on the addition of a monomer unit in an active site (C^I*^, C^II*^). Such an addition of monomer occurs through the coordination and subsequent insertion of the monomer in the active site, giving rise to the propagating polymer chain (*P_n_^I^*, *P_n_^II^*) of length (*n* = 1). The propagation step consists of the growth of the propagating polymer chain *P_n_*, which proceeds by successive coordination-insertion of monomers in the propagating polymer chain *P_n_*, leading to the growth of the length *n*. In addition to the chain initiation and propagation, the common coordination polymerization in neodymium-based catalysts also feature an irreversible chain transfer reaction to the cocatalyst.

The polymer chain (*P_n_^I^*, *P_n_^II^*) transfer to the alkyl-aluminum cocatalyst (chain transfer agent, *AlR_x_*) generates inactive species (inactive chains for monomer insertion located on aluminum, *P_n_Al^I^* and *P_n_Al^II^*) and regenerates new active centers (C_1_^I*^, C_1_^II*^), which can continue the initiation and propagation reaction. The type I inactive species, *P_n_Al^I^*, are considered dead polymers (*P_n_Al^I^ = D_n_^I^*) in this work; the inactive species of type II, *P_n_Al^II^*, are considered “dormant species”.

The active chain of type II (*P_n_^II^*) and dormant species (*P_m_Al^II^*) undergo a chain exchange reaction between them. The chain growth occurs only in the propagating active polymer *P_n_^II^*; when the *P_n_^II^* is transferred to *P_m_Al^II^*, the dormant chain *P_m_* is “activated” and it is converted to the propagating chain, and the active chain *P_n_^II^* that transferred on aluminum is converted to “deactivated”, known as the dormant species *P_n_Al^II^*.

The reversible chain transfer reaction between active chain and dormant species is the basis of CCTP, and this reaction allows narrow molecular weight distributions and growth of multiple polymer chains per catalyst molecule, especially when the deactivation and termination of propagating chains are negligible. This reaction step could be categorized as a catalytic degenerative chain transfer, similar to that of free-radical polymerization, a term recommend by the International Union of Pure and Applied Chemistry (IUPAC) [25].

### 3.2. Population Balance Equations (PBEs)

The PBEs were derived from the reaction mechanism shown in Figure 1, Equations (3)–(11)

(3) $$\frac{d\;\left[C^{*\;I}\right]}{dt}=-k_{in,\;I}\;\left[C^{*\;I}\right]\left[M\right]+k_{trc,\;I}\overset{N}{\underset{n=1}{\sum }}\left[P_{n}{}^{I}\right]\left[AlR_{x}\right]-k_{da1,\;I}\;\left[C^{*\;I}\right]$$

(4) $$\frac{d\;\left[C^{*\;II}\right]}{dt}=-k_{in,\;\mathrm{II}}\;\left[C^{*\;II}\right]\left[M\right]+k_{trc,\;\mathrm{II}}\overset{N}{\underset{n=1}{\sum }}\left[P_{n}{}^{II}\right]\left[AlR_{x}\right]-k_{da1,\;\mathrm{II}}\;\left[C^{*\;II}\right]$$

(5) $$\frac{d\;\left[M\right]}{dt}=-k_{in,\;I}\;\left[C^{*\;I}\right]\left[M\right]-k_{p,\;I}\overset{N}{\underset{n=1}{\sum }}\left[P_{n}{}^{I}\right]\left[M\right]-k_{in,\;II}\left[C^{*\;II}\right]\left[\;M\right]-k_{p,\;II}\overset{N}{\underset{n=1}{\sum }}\left[P_{n}{}^{II}\right]\left[M\right]$$

(6) $$\frac{d\;\left[AlR_{x}\right]}{dt}=-k_{trc,\;I}\overset{N}{\underset{n=1}{\sum }}\left[P_{n}{}^{I}\right]\left[AlR_{x}\right]-k_{trc,\;II}\overset{N}{\underset{n=1}{\sum }}\left[P_{n}{}^{II}\right]\left[AlR_{x}\right]$$

(7) $$\frac{d\;\left[P_{n}{}^{I}\right]\;}{dt}=k_{in,I}\left[C^{*\;I}\right]\left[M\right]-k_{trc,\;I\;}\left[P_{n}{}^{I}\right]\left[AlR_{x}\right]-k_{da2,I}\left[P_{n}{}^{I}\right]$$

(8) $$\frac{d\;\left[D_{n}{}^{I}\right]}{dt}=k_{da2,\;I}\;\left[P_{n}{}^{I}\right]+k_{trc,\;I\;}\left[P_{n}{}^{I}\right]\left[AlR_{x}\right]$$

(9) $$\frac{d\left[P_{n}{}^{II}\right]}{dt}=k_{in,\;II}\left[C^{*II}\right]\left[M\right]-k_{trc,\;II}\left[P_{n}{}^{II}\right]\left[AlR_{x}\right]-\left(k_{tr}+k_{tr1}\right)\left[P_{n}{}^{II}\right]\overset{N}{\underset{m=1}{\sum }}\left[AlP_{m}{}^{II}\right]\;+\left(k_{tr1}+k_{tr}\right)\left[P_{m}{}^{II}\right]\overset{N}{\underset{n=1}{\sum }}\left[Al\;P_{n}{}^{II}\right]-k_{da2,II}\left[P_{n}{}^{II}\right]$$

(10) $$\frac{d\left[AlP_{n}{}^{II}\right]}{dt}=k_{trc,\;II}\overset{N}{\underset{n=1}{\sum }}\left[P_{n}{}^{II}\right]\left[AlR_{x}\right]+\left(k_{tr1}+k_{tr}\right)\overset{N}{\underset{m=1}{\sum }}\left[P_{m}{}^{II}\right]\left[Al\;P_{n}{}^{II}\right]\;-\left(k_{tr}+k_{tr1}\right)\;\overset{N}{\underset{n=1}{\sum }}\left[P_{n}{}^{II}\right]\left[Al\;P_{m}{}^{II}\right]$$

(11) $$\frac{d\left[D_{n}{}^{II}\right]}{dt}=k_{da2,II}\;\left[P_{n}{}^{II}\right]$$

### 3.3. Method of Moments

The method of the moments is a versatile tool for the modeling of the polymerization kinetics and the estimation of the unreported kinetic parameters in the literature. More details about the method can be found in several texts [26,27,28,29].

First, the definitions of the moments for each polymer species are presented; two types of polymers (type I and II) have been distinguished during the experiments, as is supported by the results. Therefore, the kinetic model needs to predict the characteristics for both types of populations.

The *k*-th moment for the active and dead polymer of type I are described in Equations (12) and (13), respectively.

(12) $$\mu_{k}{}^{I}=\overset{N}{\underset{n=1}{\sum }}n^{k}\left[P_{n}{}^{I}\right]$$

(13) $$\nu_{0}{}^{I}=\overset{N}{\underset{n=1}{\sum }}n^{k}\left[D_{n}{}^{I}\right]$$

The *k*-th moment for the active, dormant and dead polymers of type II are defined in Equations (14)–(16), respectively.

(14) $$\mu_{k}{}^{II}=\overset{N}{\underset{n=1}{\sum }}n^{k}\left[P_{n}{}^{II}\right]$$

(15) $$\beta_{k}{}^{II}=\overset{N}{\underset{n=1}{\sum }}n^{k}\left[P_{n}Al^{II}\right]$$

(16) $$\nu_{0}{}^{II}=\overset{N}{\underset{n=1}{\sum }}n^{k}\left[D_{n}{}^{II}\right]$$

The zeroth moments are derived from the PBEs, Equations (3)–(11), after some straightforward mathematical manipulations. The zeroth moments for the active (or propagating) polymer species and dead polymers for type I are presented in Equations (17) and (18), respectively. The zeroth moments for the active, dormant and dead polymer species of type II are written in the Equations (19)–(21), respectively.

Polymer of type I

(17) $$\frac{d\mu_{0}{}^{I}}{dt}=k_{in,\;I}\left[C^{*\;I}\right]\left[M\right]-k_{trc,\;I}\mu_{0}{}^{I}\left[AlR_{x}\right]-k_{da2,\;I}\mu_{0}{}^{I}$$

(18) $$\frac{d\nu_{0}{}^{I}}{dt}=k_{da2,\;I}\mu_{0}{}^{I}+k_{trc,\;I}\mu_{0}{}^{I}\left[AlR_{x}\right]$$

Polymer of type II

(19) $$\frac{d\mu_{0}{}^{II}}{dt}=k_{in,\;\mathrm{II}}\left[C^{*\;II}\right]\left[M\right]-k_{trc,\;II}\mu_{0}{}^{II}\left[AlR_{x}\right]-k_{da2,\;\mathrm{II}}\mu_{0}{}^{II}$$

(20) $$\frac{d\beta_{0}{}^{II}}{dt}=k_{trc,\;\mathrm{II}}\;\mu_{0}{}^{II}\left[AlR_{x}\right]$$

(21) $$\frac{d\nu_{0}{}^{II}}{dt}=k_{da2,\;\mathrm{II}}\;\mu_{0}{}^{II}$$

The first moments are presented in Equations (22)–(26)

Polymer of type I

(22) $$\frac{d\mu_{1}{}^{I}}{dt}=k_{in,\;I}\left[C^{*\;I}\right]\left[M\right]+k_{p,\;I}\left[M\right]\mu_{0}{}^{I}-k_{trc,\;I\;}\mu_{1}{}^{I}\left[AlR_{x}\right]-k_{da2,\;I}\mu_{1}{}^{I}$$

(23) $$\frac{d\nu_{1}{}^{I}}{dt}=k_{da2,\;I}\left[\mu_{1}{}^{I}\right]+k_{trc,\;I}\mu_{1}{}^{I}\left[AlR_{x}\right]$$

Polymer of type II

(24) $$\frac{d\mu_{1}{}^{II}}{dt}=k_{in,\;\mathrm{II}}\left[C^{*II}\right]\left[M\right]+k_{p,\;\mathrm{II}}\left[M\right]\mu_{0}{}^{II}-k_{trc,\;\mathrm{II}\;}\mu_{1}{}^{II}\left[AlR_{x}\right]-\left(k_{tr1}+k_{tr}\right)\left(\mu_{1}{}^{II}\beta_{0}{}^{II}\right)+\left(k_{tr}+k_{tr1}\right)\left(\mu_{0}{}^{II}\;\beta_{1}{}^{II}\right)-k_{da2,\;\mathrm{II}}\;\mu_{1}{}^{II}$$

(25) $$\frac{d\beta_{1}{}^{II}}{dt}=k_{trc,\;\mathrm{II}}\;\mu_{1}{}^{II}\left[AlR_{x}\right]+\left(k_{tr1}+k_{tr}\right)\left(\mu_{1}{}^{II}\beta_{0}{}^{II}\right)-\left(k_{tr}+k_{tr1}\right)\left(\mu_{0}{}^{II}\;\beta_{1}{}^{II}\right)$$

(26) $$\frac{d\;\nu_{1}{}^{II}}{dt}=k_{da2,\;\mathrm{II}}\mu_{1}{}^{II}$$

Finally, the second moments are derived and they are presented in Equations (27)–(31)

Polymers of type I

(27) $$\frac{d\mu_{2}{}^{I}}{dt}=k_{in,\;I}\left[C^{*\;I}\right]\left[M\right]+k_{p,\;I}\left[M\right]\left(2\;\mu_{1}{}^{I}+\mu_{0}{}^{I}\right)-k_{trc,\;I\;}\mu_{2}{}^{I}\left[AlR_{x}\right]-k_{da2,\;I}\mu_{2}{}^{I}$$

(28) $$\frac{d\nu_{2}{}^{I}}{dt}=k_{da2,\;I}\;\left[\mu_{2}{}^{I}\right]+k_{trc,\;I}\mu_{2}{}^{I}\;\left[AlR_{x}\right]$$

Polymers of type II

(29) $$\frac{d\;\mu_{2}{}^{II}}{dt}=k_{in,\;\mathrm{II}}\left[C^{*III}\right]\left[M\right]+k_{p,\;\mathrm{III}}\;\left[M\right]\left(2\;\mu_{1}{}^{II}+\mu_{0}{}^{II}\right)-k_{trc,\;\mathrm{II}}\mu_{2}{}^{II}\left[AlR_{x}\right]\;-\left(k_{tr}+k_{tr1}\right)\left(\mu_{2}{}^{II}\beta_{0}{}^{II}\right)+\left(k_{tr1}+k_{tr}\right)\left(\mu_{0}{}^{II}\beta_{2}{}^{II}\right)-k_{da2,\;\mathrm{II}}\mu_{2}{}^{II}$$

(30) $$\frac{d\;\beta_{2}{}^{II}}{dt}=k_{trc}\;\mu_{2}{}^{II}\left[AlR_{x}\right]+\left(k_{tr1}+k_{tr}\right)\left(\mu_{2}{}^{II}\beta_{0}{}^{II}\right)-\left(k_{tr}+k_{tr1}\right)\;\left(\mu_{0}{}^{II}\;\beta_{2}{}^{II}\right)$$

(31) $$\frac{d\;\nu_{2}{}^{II}}{dt}=k_{da2,\;\mathrm{II}}\left[\mu_{2}{}^{II}\right]$$

*M_n_* and *M_w_* were calculated for by Equations (32) and (33), respectively, for polymers of type I, and by Equations (34) and (35) for polymers of type II. In addition, the overall *M_n_* and *M_w_* can be calculated, using Equations (36) and (37):

Polymers of type I

(32) $$M_{n}{}^{I}=\frac{\mu_{1}{}^{I}+\nu_{1}{}^{I}}{\mu_{0}{}^{I}+\nu_{0}{}^{I}}\left(MW_{Mon}\right)$$

(33) $$M_{w}{}^{I}=\frac{\mu_{2}{}^{I}+\nu_{2}{}^{I}}{\mu_{1}{}^{I}+\nu_{1}{}^{I}}\left(MW_{Mon}\right)$$

Polymers of type II

(34) $$M_{n}{}^{II}=\frac{\mu_{1}{}^{II}+\beta_{1}{}^{II}+\nu_{1}{}^{II}}{\mu_{0}{}^{II}+\beta_{0}{}^{II}+\nu_{0}{}^{II}}\left(MW_{Mon}\right)$$

(35) $$M_{w}{}^{II}=\frac{\mu_{2}{}^{II}+\beta_{2}{}^{II}+\nu_{2}{}^{II}}{\mu_{1}{}^{II}+\beta_{1}{}^{II}+\nu_{1}{}^{II}}\left(MW_{Mon}\right)$$

Overall:

(36) $$M_{n}=\frac{\mu_{1}{}^{I}+\nu_{1}{}^{I}+\mu_{1}{}^{II}+\beta_{1}{}^{II}+\nu_{1}{}^{II}}{\mu_{0}{}^{I}+\nu_{0}{}^{I}+\mu_{0}{}^{II}+\beta_{0}{}^{II}+\nu_{0}{}^{II}}\left(MW_{Mon}\right)$$

(37) $$M_{w}=\frac{\mu_{2}{}^{I}+\nu_{2}{}^{I}+\mu_{2}{}^{II}+\beta_{2}{}^{II}+\nu_{2}{}^{II}}{\mu_{1}{}^{I}+\nu_{1}{}^{I}+\mu_{1}{}^{II}+\beta_{1}{}^{II}+\nu_{1}{}^{II}}\left(MW_{Mon}\right)$$

The dispersity of the system is calculated by Equation (38)

(38) $$Đ=\frac{M_{w}}{M_{n}}$$

The “experimental” average number of polymer chains (*Np_Exp_*) produced by a single primary metal atom is calculated using Equation (39)

(39) $$Np_{Exp}=\frac{M_{n\;Theo}}{M_{n\;Exp}}$$

where

(40) $$M_{n\;Theo}=\frac{{\left[M\right]}_{0}}{{\left[C^{*I}\right]}_{0}+{\left[C^{*II}\right]}_{0}}\left(MW_{Mon}\right)\left(X\right)$$

In addition, the theoretical average number of polymer chains (*Np_Theo_*) produced by a single primary metal atom is calculated by the Equation (41)

(41) $$Np_{Theo}=\frac{M_{n\;Theo}}{M_{n\;Model}}$$

where *M_n_*_*Model*_ was calculated using Equation (36)

The End-Group Functionality (EGF) [30] for polymer chains of Type *II* is calculated using Equation (42)

(42) $$EGF_{II}=\frac{\mu_{0}{}^{II}+\beta_{0}^{II}}{\mu_{0}{}^{II}+\beta_{0}^{II}+\nu_{0}{}^{II}}$$

### 3.4. Optimization Strategy for the Parameter Estimation

In this section, the objective is the estimation of the kinetic parameters used in the moment equations (Equations (17)–(31)) for the CCTP process. Two polymer populations can be distinguished, described as Type I and II, and it is assumed that their kinetic behaviors can be completely separated, as proposed in the polymerization mechanism (Figure 1). Therefore, there is no interconnectivity between the polymer species of Type I and species of Type II. The kinetic parameters involved in the evolution of the polymers of Type II, those governing the CTTP process, was adjusted by an optimization methodology. The minimization of sum of squared errors (SSE) between the model predicted values and the experimental values was carried out. The objective function is the Equation (43):

(43) $$min\overset{n}{\underset{i=1}{\sum }}\left\{{\left(\frac{\left[M^{P}\right]\left(t_{i}\right)-\left[M^{Exp}\right]\left(t_{i}\right)}{\left[M^{Exp}\right]\left(t_{i}\right)}\right)}^{2}+{\left(\frac{M_{n}^{P}\left(t_{i}\right)-M_{n}^{Exp}\left(t_{i}\right)}{M_{n}^{Exp}\left(t_{i}\right)}\right)}^{2}\;+{\left(\frac{M_{w}^{P}\left(t_{i}\right)-M_{w}^{Exp}\left(t_{i}\right)}{M_{w}^{Exp}\left(t_{i}\right)}\right)}^{2}\right\}\;\mathrm{s.t.}\;k_{in,II},k_{p,II},\;k_{trc,II},k_{tr},\;k_{da1,II},k_{da2,II},>0\;k_{in,II},k_{p,II},\;k_{trc,II},k_{tr},\;k_{da1,II},k_{da2,II}\;\in R^{n}$$

where $\left[M^{P}\right]\left(t_{i}\right)$ denotes the predicted value of the remaining monomer at the time *i,* calculated using Equation (5) (involving only the terms of species II)*;* $\left[M^{Exp}\right]\left(t_{i}\right)$ is the experimentally measured value of the remaining monomer at the time *i*; $M_{n}^{P}\left(t_{i}\right)$ and $M_{w}^{P}\left(t_{i}\right)$ are the predicted values of the number and weight average molecular weights at time *i*, respectively, calculated by Equations (34) and (35) in the model; and $M_{n}^{exp}\left(t_{i}\right)$ and $M_{w}^{exp}\left(t_{i}\right)$ are the experimental data of the number and weight average molecular weights at time *i*.

The coefficient of determination (*R_j_^2^*) was also calculated for each characteristic *j* using Equation (44), [31] being *j* = [*M*], *M_n_* and *M_w._*

(44) $$R_{j}^{2}=1-\frac{SS_{res}^{j}}{SS_{tot}^{j}}$$

where

(45) $$SS_{Res}^{J}=\overset{n}{\underset{i=1}{\sum }}\frac{{\left(\varphi_{j}^{P}\left(t_{i}\right)-\varphi_{j}^{Exp}\left(t_{i}\right)\right)}^{2}}{n-1}$$

(46) $$SS_{Tot}^{J}=\overset{n}{\underset{i=1}{\sum }}\frac{{\left(\varphi_{j}^{P}\left(t_{i}\right)-{\hat{\varphi }}_{j}^{Exp}\right)}^{2}}{n-1}$$

where $\varphi_{j}^{Exp}\left(t_{i}\right)$ is the experimental value of the characteristic *j* ([*M*], *M_n_* or *M_w_*) at the time *i*, $\varphi_{j}^{P}\left(t_{i}\right)$ is the predicted value of the characteristic *j* at time i, ${\hat{\varphi }}_{j}^{Exp}$ is the average experimental value for a specific characteristic, and *n* is the total number of data for each characteristic.

Additionally, the standard deviation (*S*) is defined in Equation (47)

(47) $$S=\sqrt{\overset{n}{\underset{i=1}{\sum }}\frac{{\left(\varphi_{j}^{P}\left(t_{i}\right)-\varphi_{j}^{Exp}\left(t_{i}\right)\right)}^{2}}{n-1}}$$

### 3.5. Numerical Aspects and Equipment

The ODE systems for the moments equations were solved by MATLAB R2019a code using ode23s, which is a solver for stiff differential equations, based on a modified Rosenbrock formula of order 2. Ode23s permits relative tolerances or problems with solutions that change rapidly, due to it being a single-step solver with an evaluation of the Jacobian during each step of the integration [32]. In addition, the tool *Fmincon* was used to find the minimum of the objective function, Equation (42); an easy implementation of this robust optimization tool is the principal characteristic [33]. In all the calculations a standard laptop computer running at 2.10 GHz was used, with 12 GB of RAM, Processor Intel^®^ Core™ i3-10110U CPU @ 2.10 GHz 2.59 GHz.

## 4. Results and Discussion

### 4.1. Polymerizations

The experimental design of the CCTP reactions is shown in Table 1, the ratio [*M*]_0_/[*Nd*]_0_ varies in three levels and the temperature is analyzed also in three levels for the ratio [*M*]_0_/[*Nd*]_0_ = 660. The ratios [*AlR_x_*]_0_/[*Nd*]_0_ and [*R_H_Cl_2_*]_0_/[*Nd*]_0_ are maintained constants.

**Figure 2:**
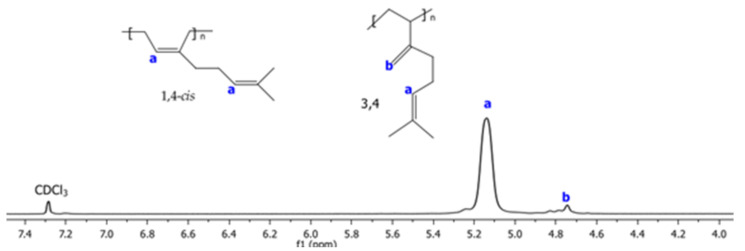
^1^H NMR spectra using CDCl3 as solvent of poly(β-myrcene) for Exp. 2.

**Figure 3:**
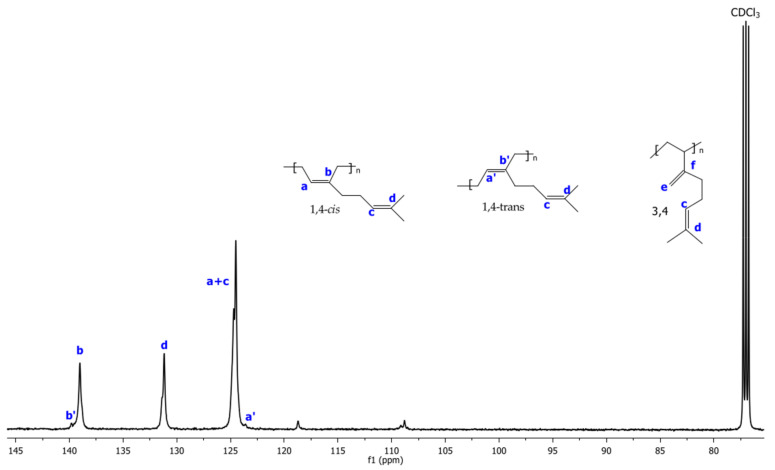
^13^C NMR spectra using CDCl3 as solvent of poly(β-myrcene) for Exp. 2.

The ^1^H and ^13^C NMR spectra corresponding to Exp 2 are illustrated in Figure 2 and Figure 3, respectively. The structural isomers 3,4- and 1,4- (*cis* + *trans*) were determined by ^1^H NMR spectra integrating signals in the region of 4.7 to 5.3 ppm, respectively (Figure 2). The *cis*/*trans* ratio was estimated in the ^13^C NMR spectra (proton gated decoupling no-NOE experiments) [34], by integration of the olefinic group signals (Figure 3). The resultant structural content in the poly(β-myrcene) estimated by ^1^H NMR is 6% of 3,4- and 94% of 1,4-content. Additionally, the stereoisomerism quantification for ^13^C NMR results in 92% of 1,4-*cis* and 8% of 1,4-*trans*. Furthermore, the variation of the catalytic system used for experimental data does not present an important effect on the polymer microstructure, favoring the high generation of c*is* isomers (90–95%), attributed to the steric volume of the alkyl groups attached to the double bond of the isoprene unit of the monomer, coordinating in η^4^ to the catalytic center [11].

**Figure 4:**
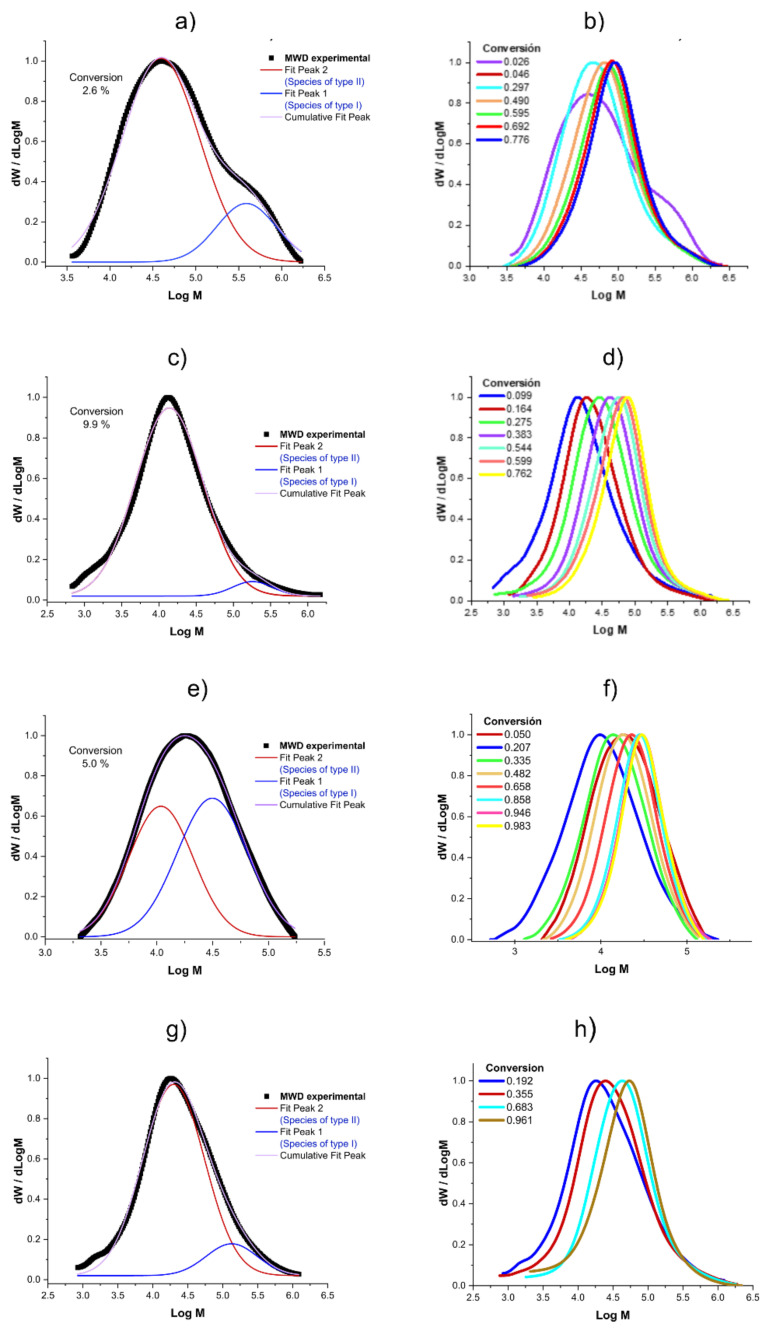
Molecular weight distributions: (**a**) MWD and the deconvolution for Exp. 1 at 2.6% of conversion; (**b**) Evolution of the MWD in conversion for Exp. 1; (**c**) MWD and the deconvolution for Exp. 3 at 9.9% of conversion; (**d**) Evolution of the MWD in conversion for Exp. 3; (**e**) MWD and the deconvolution for Exp. 4 at 5% of conversion; (**f**) Evolution of the MWD in conversion for Exp. 4; (**g**) MWD and the deconvolution for Exp. 5 at 1.9% of conversion; and (**h**) Evolution of the MWD in conversion for Exp. 5. Figure S1 in the Supporting Information shows the plots for Exp 2.

As can be seen in Figure 4a,c,e,g for Exp. 1, 3, 4 and 5, respectively, in the initial stages, a conventional coordination polymerization with a ternary Ziegler–Natta Nd-based catalyst system takes place, wherein the concentration of DIBAH, which is also the chain transfer agent, is high, so the chain transfer is fast, thereby producing a broad molecular weight distribution (MWD) with high MWs; at this point, the conversion and *Np* are low (conversion = 2.6% with *Np* = 0.1 for Exp. 1, conversion = 5% with *Np* = 0.8 for Exp. 4). However, as the conversion increases the competition between the irreversible chain transfer and reversible chain transfer begins to be relevant, especially since the DIBAH concentration decreases markedly, together with the chain transfer. This can be evidenced by an increase in *Np*, which reaches a constant value of *Np* = 1.1 and 4 near 50% of conversion for Exp. 1 and 4, which implies clearly that the reversible chain transfer becomes the dominant reaction process. Under this scenario, the CCTP regime controls the polymerization behavior, wherein the chain growth takes place only through the active centers of the Nd catalyst. While the polymer chains are transferred to the respective centers, the chains rest in a dormant state, where chain termination is suppressed. Nevertheless a dormant chain can be activated to become an active chain via reversible chain transfer, therefore the polymer chains are synchronously growing and narrow MWD are produced [9] which implies an unimodal MWD with a shoulder at high MWs (Figure 4b,d,f,g). F Therefore, the bimodal MWD produced at low conversion levels is attributed to the presence of two populations, and it disappears at high conversions due to the higher concentration of the dormant species generated in the CCTP regime than that of the dead polymer produced in a conventional CCP, which appear as a shoulder with high MWs for all the experiments.

### 4.2. Modeling and Simulations

According to Friebe et al. [35], the ratio [*R_H_Cl_2_*]_0_/[*Nd*]_0_ has a significant impact on the *k_app_* estimated for the butadiene polymerization initiated by the ternary Ziegler–Natta catalyst system comprised of NdV_3_, DIBAH and ethyl aluminum sesquichloride (EASC) at 60 °C. In this part of the work, the selected experiments maintain the ratios of [*R_H_Cl_2_*]_0_/*[Nd]*_0_ = 1 and [*AlR_x_*]_0_/[*Nd*]_0_ = 20 as constants, in order to keep a low degree of freedom, and only to study the impact of the ratio [*M*]_0_/[*Nd*]_0_ and the temperature.

In the next sections, the estimation of the effective concentration of DIBAH acting as control agent and the kinetic rate constants are carried out, using the optimization tool. The experiments selected (Exp 1–5) are analyzed under theoretical modeling. Afterward, a set of the kinetic rate constants is estimated by the optimization methodology.

#### 4.2.1. Estimation of [AlR_x_]_0_

It is well known that the DIBAH has a dual function during the polymerization, acting as a chain transfer agent for the control of the average molecular weights and narrow distributions, and also acting as a scavenger of impurity moiety, e.g., the moisture and carboxylic acids [36]. That means that the effective concentration of DIBAH involved in the formation of dormant chains (*P_n_Al^II^*, in Figure 1) is somewhat uncertain and it can depend on the unavoidable presence of impurities in the reaction medium and on the particularly employed reaction conditions [16].

**Figure 5:**
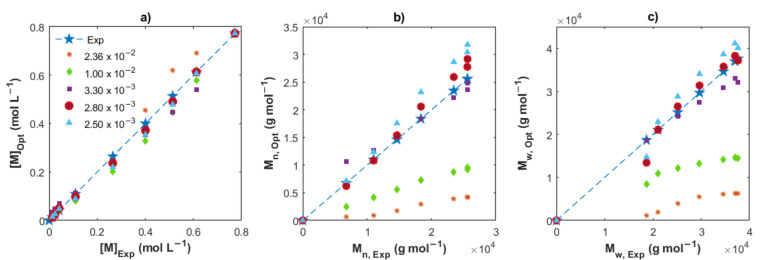
Comparison between the experimental data and the optimized values for Exp. 2, varying [*AlR_x_*]_0_. (**a**) Remaining monomer; (**b**) *M_n_*; and (**c**) *M_w_*.

To estimate the effective concentration of DIBAH which takes place in the chain transfer step, Cavalcante de Sá et al. [16] used a reconciled amount of DIBAH; we understand by a reconciled amount, an equivalent proportion related with *Np*. This procedure was feasible because no deactivation reaction was considered in the reaction mechanism. However, there are chain deactivation reactions involved in our proposed scheme (Figure 1), so the assumptions to calculate the effective concentration of DIBAH used as the chain transfer agent made by Cavalcante de Sá et al. are not valid in this context. In this work, as a first approximation, we estimated the effective concentration of DIBAH (*[AlR_x_]*_0_) using several values as input of the optimization program, from the total concentration considered in the recipes to low levels. In Figure 5 the experimental measurements ([*M*], *M_n_* and *M_w_*) are plotted versus the optimal values obtained by the algorithm for Exp. 4, varying the [*AlR_x_*]_0_ from 2.36 × 10^−2^ to 2.5 × 10^−3^ mol L^−1^. While high values of [*AlR_x_*]_0_ resulted in strong deviations in *M_n_* and *M_w_* principally, the value of [*AlR_x_*]_0_ = 2.8 × 10^−3^ mol L^−1^ provided the best fit with the lowest value of the SSE. Using a high [*AlR_x_*]_0_, the activation state of the chains is reduced, predominating the dormant state in the polymerization, and the number of monomers added to the propagating chains becomes less; therefore, the *M_n_* and *M_w_* is also low. The closest match between experimental and simulated values for the set of Exps. 1, 2 and 3 is found for the resulting in a mean value of 1.2 × 10^−3^ mol L^−1^. The values found for Exps. 4 and 5 are 2.8 × 10^−3^ and 3.5 × 10^−3^ mol L^−1^, respectively. The percent of the *[AlR_x_]_0_* regarding the initial concentration of DIBAH are 5.08, 16.09 and 11.95%, which are in the same interval reported by Cavalcante de Sá et al. (from 10 to 40%) for the isoprene CCTP experiments using another initial reagent concentrations ([*RHCl_2_*]_0_/[*Nd*]_0_ = 0.5) and temperatures [16]. As mentioned previously, the termination steps, the operating conditions and the reagent ratios considered in this work do not allow a strict comparison with that work, but we could highlight the low effective concentration of DIBAH involved in the CCTP for both systems.

#### 4.2.2. Estimation of Kinetic Rate Constants

As described in Section 3.1, Section 3.4 and Section 4.1, two populations of polymer chains are distinguished during the β-myrcene polymerization: (i) the polymer of Type I only at the beginning with a fast polymerization rate, which is quickly consumed in the first stage; and (ii) the polymer of Type II, which governs the CCTP process, presenting a slower polymerization rate and homogeneous chain lengths. The kinetic behavior of these types of polymers is decoupled; hence, the optimization methodology to estimate the kinetic rate constants are only used for polymer Type II. The experimental points at short reaction times are negligible, generally below 1000 s, and it is considered that the rest of the experimental data are not affected by the low concentration of the Type I polymer.

The optimization implemented in the kinetic modeling of the polymerization minimizes the objective function, Equation (43), by searching for the best values of the kinetic rate constants (*k*’s). The relative squared difference (SSE) takes the contribution of three measured characteristics, such as the monomer conversion, *M_n_*, and *M_w_* of the estimated values and the experimental data. A set of guess values of the kinetic rate constants are required to initialize the algorithm. These guess values used as the input of the optimization algorithm were initially estimated by a manual fitting.

**Figure 6:**
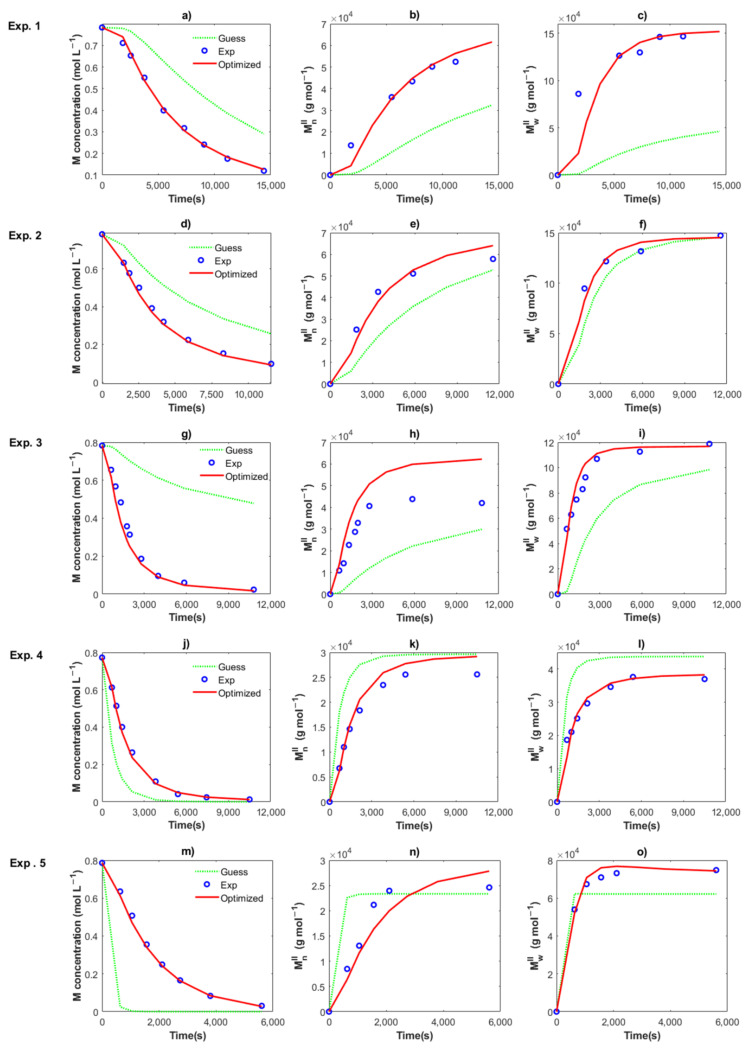
Comparison between the experimental data (circles), the solution using the guess values (dotted lines) and the solution using the optimized values (red lines) for Exps. 1 (**a**–**c**), Exps. 2 (**d**–**f**), Exps. 3 (**g**–**i**), Exps. 4 (**j**–**l**), and Exps. 5 (**m**–**o**). Remaining monomer (**a**,**d**,**g**,**j**,**m**), *M_n_* (**b**,**e**,**h**,**k**,**n**) and *M_w_* (**c**,**f**,**i**,**l**,**o**).

Figure 6 shows the optimization results of the kinetic rate constant for Exp. 1 to 5. An excellent fit between the experimental data (symbols) and the optimized solution (continuous lines) for the three characteristics with the values of *k_in,II_, k_p,II_, k_trc,II_, k_tr,II_, k_da1,II_* and *k_da2,I_* are obtained. The optimized values for the five experiments are shown in Table 2. The enlaced time expended for the program was around 6–7 s, resulting in the minimization of the SSE, from high values for the guess value (dotted lines) to low values for the optimized value. Therefore, a very efficient routine was programmed, which could be used for the estimation of parameters in other polymerization techniques. Local minimums were found during the procedure, but the lower and upper bounds of the kinetic parameters were changed to find the minimum SSE value. For the series of Exp. 1, 2 and 3 with the same recipe, and only changing the temperature at 50, 60 and 70 °C, the lower bounds were set to the optimized value of the kinetic rate constant at a lower temperature, which was previously calculated; *i.e.,* the optimized values for Exps. 1 and 2 were used as lower bounds for Exps. 2 and 3, respectively.

The optimized values were used in the solution of the full ODE system, namely Equations (1)–(4) and (15)–(29), and the values for *k_in,I_, k_p,I_, k_trc,I_, k_da1,I_* and *k_da2,I_* for the polymer of Type I were estimated by fitting the model solutions and the experimental data. It ought to be mentioned that for Exps. 1, 2 and 5 the experimental data at short reaction times were not obtained and the parameters for the species of Type I were only estimated by fitting the model to the experimental conversion profiles for simplicity. Another method for data collection could be recommended, such as online NMR and online GPC techniques, to increase the number of experimental data and improve the estimation of those kinetic rate parameters, being the moderate operating pressure the principal inconvenience.

**Figure 7:**
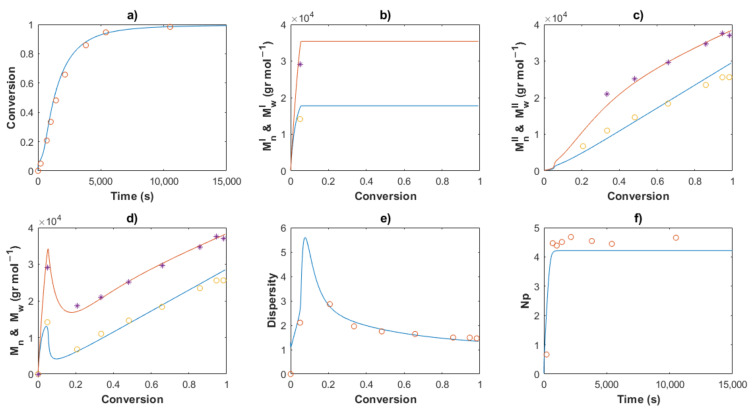
Comparison between the experimental data (symbols) and the model solutions (lines) for Exp 4: (**a**) Conversion profiles, (**b**) *M_n_^I^* and *M_w_^I^ versus* conversion, (**c**) *M_n_^II^* and *M_w_^II^ versus* conversion, (**d**) *M_n_* and *M_w_ versus* conversion, (**e**) Dispersity, (**f**) *Np* versus time.

**Figure 8:**
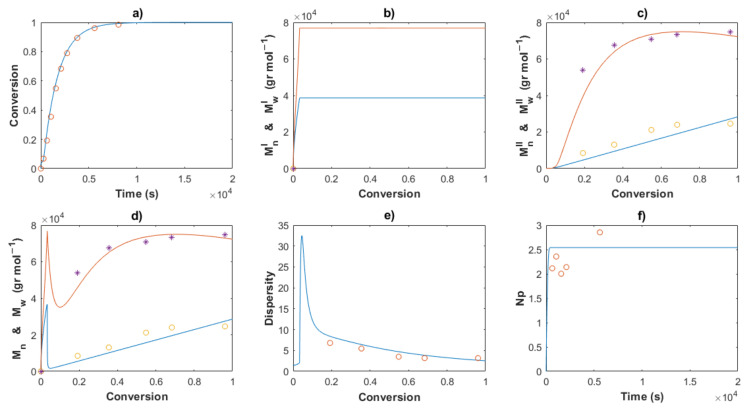
Comparison between the experimental data (symbols) and the model solutions (lines) for the Exp 5: (**a**) Conversion profiles, (**b**) *M_n_^I^* and *M_w_^I^ versus* conversion, (**c**) *M_n_^II^* and *M_w_^II^ versus* conversion, (**d**) *M_n_* and *M_w_ versus* conversion, (**e**) Dispersity, (**f**) *Np versus* time.

Table 2 shows the values for the kinetic parameters obtained after this step for all the cases and Figure 7 and Figure 8 show a comparison between the experimental data (symbols) and the solution of the mathematical model (lines) for Exps. 4 and 5, respectively. An excellent description of the conversion profiles, *M_n_^I^*, *M_w_^I^* for the polymer of Type I, *M_n_^II^*, *M_w_^II^* for the polymer of Type II, and *M_n_*, *M_w_* for overall measurements, dispersities and *Np* was obtained for all cases. According to Hustad et al. [36], the actual β-myrcene CTTP can be categorized as a semireversible chain transfer process, since the condition *k_trc,II_* > *k_tr,II_* holds true for all cases. Additionally, the coefficient of determination, Equation (44), and the standard deviation (*S*), Equation (47), were calculated for the corresponding data of [*M*], *M_n_* and *M_w_*, and the values are shown in Table 3. The profiles of [*M*] present values of *R^2^* around the unit and low values of *S* for all the experiments, attributable to the higher quantity of experimental measurements and the good fit. The values *R^2^* and *S* also show a good fit for the other two characteristics, Exp. 2 being the better for *M_n_*, and Exp. 4 being the best fit for *M_w_*.

The kinetic rate constants are very susceptible to change with the ratio [*M*]_0_/[*Nd*]_0_, as observed in the values estimated for experiments at the same temperature (Exps. 2, 4 and 5) in Table 2.

**Figure 9:**
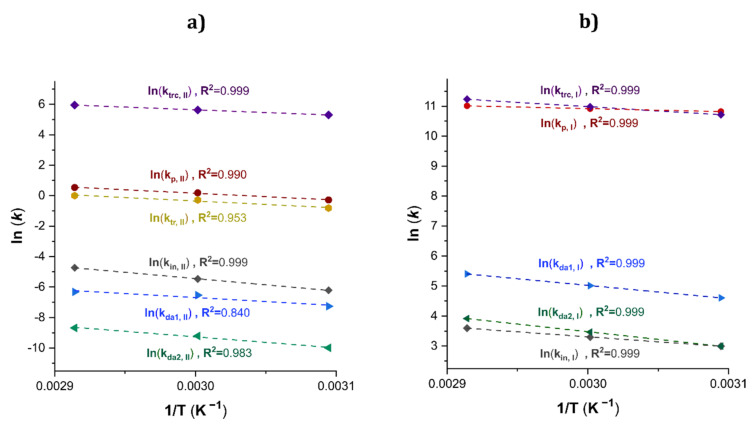
Arrhenius plot for a set of *k_j_* values for the β-myrcene CCTP for Exps. 1, 2 and 3. (**a**) Type II. (**b**) Type I.

The estimated kinetic rate coefficients of Exps. 1, 2 and 3 with increments of temperature at 50, 60 and 70 °C, respectively, were used in the calculus of the activation energy (*E_A_*) and the steric factor (also known as the pre-exponential factor) (*A*_0_). Figure 9a depicts an Arrhenius plot (ln(*k_j_*) *versus* T^−1^) and the results for the linear regression for all the *k_j_* values of the Type II with good values of *R^2^.*

The following expression for the kinetic rate coefficients were calculated

(48) $$k_{in,II}\left({L\;\mathrm{mol}}^{-1}s^{-1}\right)=2.16\times 10^{8}\exp\left(\frac{-6.83\times 10^{4}\left({J\;\mathrm{mol}}^{-1}\right)}{RT}\right)$$

(49) $$k_{tr,II}\left({L\;\mathrm{mol}}^{-1}s^{-1}\right)=6.22\times 10^{5}\exp\left(\frac{-3.80\times 10^{4}\left({J\;\mathrm{mol}}^{-1}\right)}{RT}\right)$$

(50) $$k_{da1,II}\left(s^{-1}\right)=8.96\times 10^{3}\exp\left(\frac{-4.38\times 10^{4}\left({J\;\mathrm{mol}}^{-1}\right)}{RT}\right)$$

(51) $$k_{da2,II}\left(s^{-1}\right)=2.71\times 10^{5}\exp\left(\frac{-6.04\times 10^{4}\left({J\;\mathrm{mol}}^{-1}\right)}{RT}\right)$$

where *R* = 8.314 J mol^−1^ K^−1^ and absolute temperature (*T*) is given in Kelvin (*K*).

Cavalcante de Sá et al. [16] estimated the kinetic rate constants for the solution CCTP of isoprene, initiated by Ziegler–Natta catalyst (using *Nd* compound, DIBAH and *R_H_Cl_2_*) for isothermal polymerization at 60 and 70 °C in a ratio [*Mon*]_0_/[*Nd*]_0_ = 20 and [*R_H_Cl_2_*]_0_/[*Nd*]_0_ = 0.5. A model for a single-site system was developed and the value of the pre-exponential factor and the activation energy were *A*_0_ *(k_p_*) = 4.54 × 10^2^ L mol^−1^ s^−1^ and *E_a_ (k_p_*) = 2.14 × 10^4^ J mol^−1^ for the *k_p_*, and *A*_0_ (*k_trc_*) = 7.65 × 10^6^ L mol^−1^ s^−1^ and *E_a_*(*k_trc_*) = 4.84 × 10^4^ J mol^−1^ for the *k_trc_*. Those values are similar to the estimated parameters in this work for the polymers of Type II, Equations (52) and (53):

(52) $$k_{p,II}\left(L\;mol^{-1}s^{-1}\right)=9.65\times 10^{5}\exp\left(\frac{-3.78\times 10^{4}\left({J\;\mathrm{mol}}^{-1}\right)}{RT}\right)$$

(53) $$k_{trc,II}\left({L\;\mathrm{mol}}^{-1}s^{-1}\right)=1.45\times 10^{7}\exp\left(\frac{-3.01\times 10^{4}\left({J\;\mathrm{mol}}^{-1}\right)}{RT}\right)$$

Additionally, the Arrhenius plot for the *k_j_* values of the polymers of Type I and the results for the linear regression for the *k_j_* values are shown in Figure 9b. The kinetic rate constants for Type I polymers were calculated, Equations (54) to (58).

(54) $$k_{in,I}\left({L\;\mathrm{mol}}^{-1}s^{-1}\right)=5.91\times 10^{5}\exp\left(\frac{-2.77\times 10^{4}\left({J\;\mathrm{mol}}^{-1}\right)}{RT}\right)$$

(55) $$k_{p,I}\left({L\;\mathrm{mol}}^{-1}s^{-1}\right)=1.32\times 10^{6}\exp\left(\frac{-8.78\times 10^{3}\left({J\;\mathrm{mol}}^{-1}\right)}{RT}\right)$$

(56) $$k_{trc,I}\left({L\;\mathrm{mol}}^{-1}s^{-1}\right)=3.19\times 10^{8}\exp\left(\frac{-2.38\times 10^{4}\left({J\;\mathrm{mol}}^{-1}\right)}{RT}\right)$$

(57) $$k_{da1,I}\left({L\;\mathrm{mol}}^{-1}s^{-1}\right)=8.78\times 10^{7}\exp\left(\frac{3.68\times 10^{4}\left({J\;\mathrm{mol}}^{-1}\right)}{RT}\right)$$

(58) $$k_{da2,I}\left({L\;\mathrm{mol}}^{-1}s^{-1}\right)=1.46\times 10^{8}\exp\left(\frac{-4.25\times 10^{4}\left({J\;\mathrm{mol}}^{-1}\right)}{RT}\right)$$

While the activation energies (E_a_ (*k_i,II_*)) for the β-myrcene CCTP (ratio [*M*]_0_/[*Nd*]_0_ = 750/1) estimated in this work are in the same order of magnitude (10^4^ J mol^−1^), the steric factors can vary by orders, from 10^3^ to 10^8^. An increase in the pre-exponential factor means that the chains enhance their mobility and the rotational degrees of freedom are less hindered; hence, the reaction for the formation of dormants leads to the higher mobility of the chains. It is striking that the activation energy for *k_p,II_* is equal to *k_tr,II_* and the difference lies in the steric factor, it being higher for the latter. However, these conclusions are based on the optimized fit of the kinetic rate constants to the experimental data, which are model-dependent parameters. The authors recommend carrying out an experimental estimation to improve/confirm the values reported in this work (e.g., Pulsed Laser Polymerization in combination with GPC (PLP–GPC) is a leading technique to determine kinetic coefficients in FRP); such an objective is outside of the scope of this investigation.

**Figure 10:**
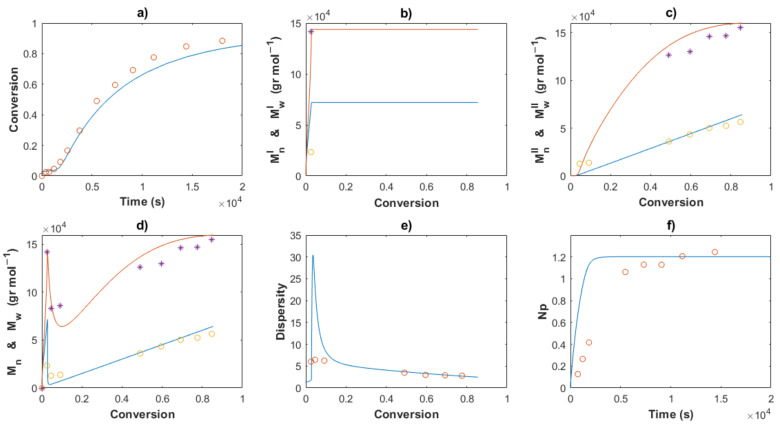
Comparison between the experimental data (symbols) and the model solutions (lines) for Exp 1: (**a**) Conversion profiles, (**b**) *M_n_^I^* and *M_w_^I^ versus* conversion, (**c**) *M_n_^II^* and *M_w_^II^ versus* conversion, (**d**) *M_n_* and *M_w_ versus* conversion, (**e**) Dispersity, (**f**) *Np versus* time.

**Figure 11:**
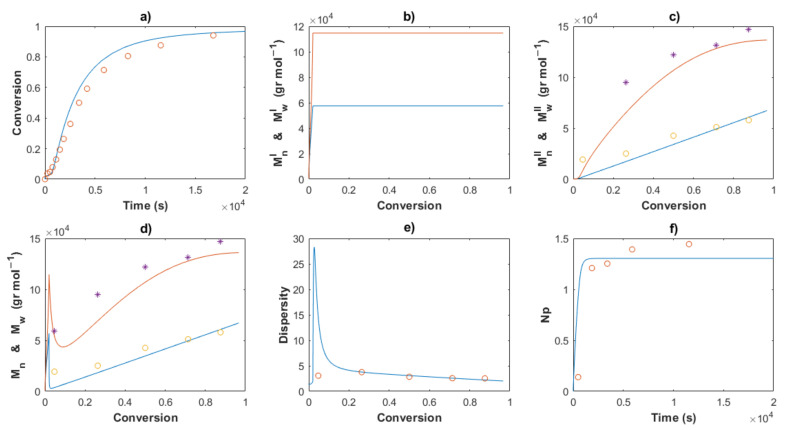
Comparison between the experimental data (symbols) and the model solutions (lines) for Exp 2: (**a**) Conversion profiles, (**b**) *M_n_^I^* and *M_w_^I^ versus* conversion, (**c**) *M_n_^II^* and *M_w_^II^ versus* conversion, (**d**) *M_n_* and *M_w_ versus* conversion, (**e**) Dispersity, (**f**) *Np versus* time.

**Figure 12:**
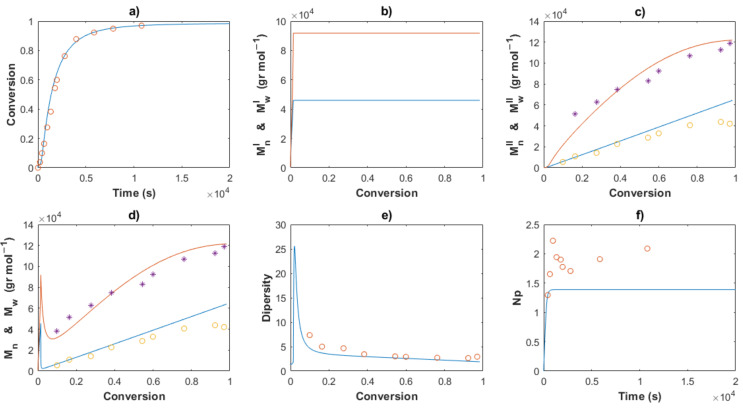
Comparison between the experimental data (symbols) and the model solutions (lines) for Exp 3: (**a**) Conversion profiles, (**b**) *M_n_^I^* and *M_w_^I^* versus conversion, (**c**) *M_n_^II^* and *M_w_^II^* versus conversion, (**d**) *M_n_* and *M_w_* versus conversion, (**e**) Dispersity, (**f**) *Np* versus time.

The Arrhenius expressions, Equations (46)–(56), were incorporated in the mathematical model in the series of [*M*]_0_/[*Nd*]_0_ = 660 for Exps. 1, 2 and 3 and the kinetic predictions are compared with the experimental data, as shown in Figure 10, Figure 11 and Figure 12, respectively. An excellent description of the experimental behavior for the three temperatures is obtained and practically the same values as those shown in Table 3 were calculated for the coefficients of determination and the standard deviations in a comparison with the experimental measurements. Incises *a* for each figure show the conversion profiles of the experimental data (points) and the simulations. It is clear that a temperature increase gives rise to a faster polymerization rate, which importantly affects the molecular characteristics as *M_n_*, *M_w_*, dispersity and *N_p_*; these are discussed in the next sections. Additionally, it is expected that the isomerism content of the polymer chains remains unaffected by the temperature change, as discussed previously.

### 4.3. Kinetic Analysis

#### 4.3.1. Polymer Chain Species

**Figure 13:**
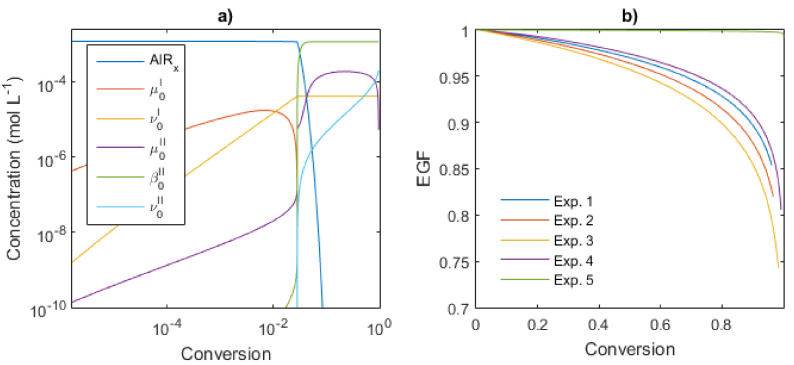
Analysis of the Kinetic behavior for the Exp 1: (**a**) Concentration profiles versus conversion, (**b**) EGF versus conversion. Operating conditions and initial concentrations are given in Table 1.

The mathematical model developed is used to study the kinetics of the reagents and the polymer species of Type I and II during the reaction; the rate constants previously estimated were used as the input of the program. First, the temperature series (Exps. 1–3) were analyzed and the total concentrations of the species are shown in Figure 13a with a log-log scale for an operating temperature of 50 °C. The kinetics of the system is divided into three stages: the first to conversion below 2% (80 s), followed by a short transition period between 2 to 3.5% (950 s) and a final equilibrium of the chain transfer for the polymers of Type II. The first stage corresponds to the growth of the polymer chains of Type I (namely *µ_0_^I^*); they reach a maximum at 0.7% (7 ms) and their concentration sharply falls to very low levels. The dead polymer chain formed during this period (*ν_0_^I^*) is analogous to a product generated by a conventional catalyst polymerization with uncontrolled monomer addition to the propagating chains. In the transition period, the total concentration of *µ_0_^I^* is exhausted and contrary *µ_0_^II^* is strongly accumulated, increasing by eight orders of magnitude, leading to a high likelihood that *P_r_* reacts with *AlRx* to form dormant chains *β_0_^II^*. Here, the polymerization rate is very slow, almost as an inhibition stage, which can be observed at short reaction times in Figure 10a. The equilibrium period is established when *β_0_^II^* reaches the plateau with a constant concentration, being *β_0_^II^* ≈ [*AlRx*]_0_. The goal of CCPT, similar to the reversible deactivation radical polymerization (RDRP), is to avoid the termination events; hence, the concentration of *ν_0_^II^* is maintained at low levels, as low as 1.5 mM, two orders of magnitude lower than *β_0_^II^*.

The kinetic behavior for Exps. 4 and 5 are as previously described, but for the latter the concentration of the dead polymer species only reaches low levels (10^−5^ mol L^−1^).

#### 4.3.2. End-Group Functionality (EGF)

The EGF concept calculates the quantification of the “livingness” degree and it is principally used in the RDRP techniques [30], wherein the termination reactions are almost negligible. According to Fan et al. [37], the termination is highly avoided in CCPT, so the EGF is high, leading to the ability of the dormant and propagating chains to react with functional groups. The styrene CCTP process and a post-reaction with carbon dioxide and an acidic water solution were carried out to produce polystyrene carboxyl acid, confirming by FTIR spectroscopy that the polymer chains preserve functionality. Unfortunately, the quantification of the EFG was not reported.

Here, the EGF was calculated for the CCTP experiments using Equation (41) and the profiles obtained are shown in Figure 13b. The change in temperature does not significantly affect the EGF (Exps. 1–3). For this series, at 80% of conversion, the EGF values are around 0.90–0.92, which means that only 8–10% of the total chains are dead products; but in the last 20% of conversion, the EGF decreases to 0.75–0.85. If the aim of the polymerization is a functionalization postreaction, one should weigh up the advantages and disadvantages between the EGF and the conversion.

On the other hand, Exp. 5 presents very high values of EGF; the termination reactions are almost avoided and therefore most of the chains preserve functionality: 0.99 at 100% of conversion. This can be attributable to the higher concentration of the catalytic system, [*M*]_0_/[*Nd*]_0_ = 533. Besides, for Exp. 5 with the lowest ratio of [*M*]_0_/[*Nd*]_0_ = 885, its EGF profile results in lower values than Exp. 5. As mentioned, the changes in two different variables in the Exp. 4 respect to the Exp. 2 or 5 (ratio [*M*]_0_/[*Nd*]_0_ and the solid content) are difficult to attribute the resulting effects to some of the two sources. Experimental validation of these results has been conducted via a functionalization postreaction and will be published in future work.

#### 4.3.3. Molecular Weights and Dispersity

The comparisons of the *M_n_*, *M_w_* and dispersity values for partial and overall predictions and experimental data are shown in Figure 7, Figure 8, Figure 10, Figure 11 and Figure 12

*M_n_*^I^ and *M_w_^I^* (incises b) present a sudden increase at the beginning of the polymerization for all cases, corresponding to the first stage, wherein a conventional coordination catalyst polymerization takes place; afterward, a constant value is calculated, since the inactivated chains of Type I (dead polymer) subsist and they do not interact with other species in the later stages. The highest value of *M_n_*^I^ is obtained with the ratio [*M*]_0_/[*Nd*]_0_ = 750.

A linear increase of *M_n_*^II^ versus conversion is seen for all the predictions (incises c) and a good description of the experimental data can be observed. This behavior confirms the good control in the addition of the monomer to the propagating chain and the polymer of Type II governs the catalytic polymerization under CCTP conditions. The higher the temperature, the higher the expected value of *M_n_^I^*. After a short period, in which the mass of polymer of Type I is greater than that of Type II, the value of *M_n_^II^* describes the overall *M_n_* (incises d).

The dispersity curves (incises e) describe the experimental data qualitatively at low conversion, presenting a maximum in the first stages due to the uncontrolled population of polymers of Type I. Then, the dispersity values continuously fall and an excellent quantitative prediction of the experimental data is seen in the plots.

#### 4.3.4. Stoichiometrical Analysis of the Polymerization

In the living polymerizations (ionic processes), the produced macromolecules can be predesigned with excellent precision, estimating the value of *M_n_* with a stoichiometrical ratio between the molar initial concentration of the monomer and the initiator, and its deviations with the experimental data are attributable to impurities and side reactions, etc. Using this ratio in CCPT, a theoretical value of *M_n_* (Equation 40) can be calculated. However, it is well-known that the ratio between the *M_n_*_theo_ and *M_n_* experimental measurements calculates the number of polymer chains produced per neodymium atom, *Np* (Equation (38)). According to Georges et al. [38], the β-myrcene polymerization via CCTP using Cp*La(BH_4_)_2_(THF)_2_ combined with Magnesium and Aluminum Alkyls results in a wide range of values of *Np*, from 2.6 to 10.7, depending on the reagent molar ratio used. In this work, the evolution of *Np versus* time is plotted for all the cases, Figure 5, Figure 6, Figure 8, Figure 9 and Figure 10, incises f, and the predictions agree with the experimental values. During the first stages of the polymerization, low values of *Np* are obtained due to fact that the [*Nd*] is higher than the number of chains generated. The total number of polymer chains is increased until it reaches the equilibrium of the process in the last stage, in which *Np* maintains a constant value for each experiment.

**Figure 14:**
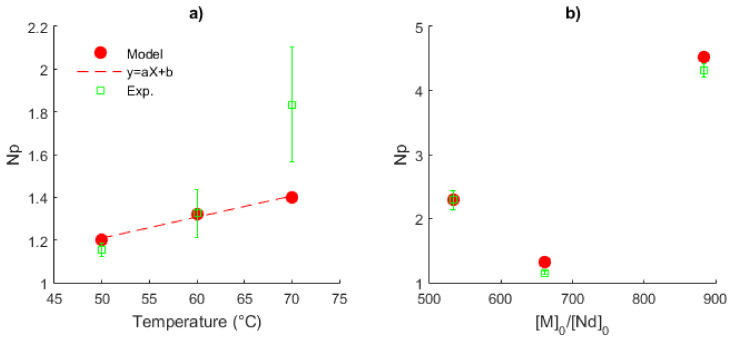
Plots of the number of polymer chains produced by the neodymium atom, Np. (**a**) Effect of the temperature, Exps. 1–3, [*M*]_0_/[*Nd*]_0_ = 660. (**b**) Effect of the ratio [*M*]_0_/[*Nd*]_0_, Exps. 2, 4 and 5 at 60 °C.

The average values of *Np* (in time), their standard deviations for the experimental data (Exps. 3–5) and the mathematical solution *versus* temperature are shown in Figure 14a for the series of Exps. 1–3. While *Np* linearly increases with the temperature for the prediction series, exhibiting a good fit of the model to low temperature, for the highest temperature (70 °C), there is a clear difference between the experimental and the predicted data. Therefore, a non-linear relationship of *Np versus* temperature is observed in the whole interval analyzed here. Additionally, the augmentation of the ratio [*M*]_0_/[*Nd*]_0_ from 533 to 885 does not show a clear tendency, Figure 14b. For the series of Exps. 3–5, a stoichiometric ratio of approximately one is obtained, analogous with the living polymerization. Under these conditions, a single chain formation in the catalytic complex is expected and predesigned molecular properties can be generated. However, for the molar ratios used in Exps. 4 and 5, the value of *Np* is incremented, giving rise to an improvement in the catalyst efficiency, as stated by Fan et al. [37].

## 5. Conclusions

The mathematical model developed for the description of the kinetics and the average molecular characteristics was used for the estimation of the set of kinetic rate constants and unknown concentrations, resulting in good agreement with the experimental data (low values of *R^2^*). Two populations were considered in the reaction mechanism and this was supported by the GPC curves. The effective concentration of DIBAH was estimated between 1.2 and 3.5 × 10^−3^ M, hence a low percentage of cocatalyst is involved in the degenerative chain transfer reaction (from 5 to 12% of the initial concentration). The estimated kinetic rate constants are strongly *Nd* concentration-dependent and, obviously, temperature-dependent. The higher pre-exponential factor value for the formation of dormant species indicated that the chains enhance their mobility and the rotational degrees of freedom are less hindered, being a key step for the control of the monomer addition to the propagating chain. The total concentration profiles showed three stages of the process: the first one involves the growth of the polymer of Type I by a conventional catalytic polymerization at low conversions, followed by a transition period with high consumption of the polymer of Type I and accumulation of the polymer of Type II, and a final period with the establishment of the equilibrium in the chain transfer reaction. In fact, the preservation of the EGF is high for all the experiments (between 75–95%) and that does not depend on the temperature; the highest value was obtained with the high concentration of DIBAH. Finally, a good prediction of *Np* for all the selected experiments was obtained, which clearly increases with the temperature, but the increase of the ratio [*M*]_0_/[*Nd*]_0_ from 533 to 885 does not show a clear condition of being directly related.

## Figures and Tables

**Table 1:** Initial concentrations, operating conditions of the CCTP reactions.

Experiment	Exp. 1	Exp. 2	Exp. 3	Exp. 4	Exp. 5
Temperature (°C)	50	60	70	60	60
[*M*]_0_/[*Nd*]_0_	660			885	533
*M*/*C-hex* (wt.%)	16			15.5	16
[*M*]_0_ (mol L^−1^)	7.83 × 10^−1^			7.73 × 10^−1^	7.86 × 10^−1^
[*Nd*]_0_ (mol L^−1^)	11.85 × 10^−4^			8.74 × 10^−4^	14.75 × 10^−4^
[*AlR_x_*]_0_ (mol L^−1^)	2.36 × 10^−2^			1.74 × 10^−2^	2.93 × 10^−2^
[*R_H_Cl_2_*]_0_ (mol L^−1^)	11.81 × 10^−4^			8.71 × 10^−4^	14.67 × 10^−4^
[*AlR_x_*]_0_/[*Nd*]_0_	20			20	20
[*R_H_Cl_2_*]_0_/[*Nd*]_0_	1			1	1

**Table 2:** Estimated kinetic rate constants for the β-myrcene CCTP.

Experiment	Exp. 1	Exp. 2	Exp. 3	Exp. 4	Exp. 5
*k_in,I_* (L mol^−1^ s^−1^)	20	27	36.45	80	15
*k_p, I_* (L mol^−1^ s^−1^)	50,000	55,000	60,500	90	20,000
*k_trc,I_* (L mol^−1^ s^−1^)	45,000	58,500	75,465	120	10,000
*k_da1,I_* (s^−1^)	100	150	222	0.16	40
*k_da2,I_* (s^−1^)	20	32	50.2	0.20	20
*k_in,II_* (L mol^−1^ s^−1^)	2 × 10^−3^	4.2 × 10^−3^	8.8 × 10^−3^	1.6 × 10^−2^	3.2 × 10^−2^
*k_p,II_* (L mol^−1^ s^−1^)	0.75	1.2	1.7	1.02	3.0
*k_da1,II_* (s^−1^)	7.00 × 10^−4^	1.45 × 10^−3^	1.80 × 10^−3^	1.25 × 10^−10^	6 × 10^−3^
*k_da2,II_* (s^−1^)	4.60 × 10^−5^	1.00 × 10^−4^	1.70 × 10^−4^	14 × 10^−4^	7 × 10^−6^
*k_trc,II_* (L mol^−1^ s^−1^)	200	280	384	30	500
*k_tr_* (L mol^−1^ s^−1^)	0.44	0.75	1	2.3	1.55

**Table 3:** Values of R^2^ and the standard deviation (S) for the model prediction of data of [*M*], *M_n_* and *M_w_*.

	Exp. 1	Exp. 2	Exp. 3	Exp. 4	Exp. 5
*R^2^_M_*	0.98	0.97	0.98	0.99	0.99
*S_M_*	0.04	0.05	0.04	0.03	0.03
*R^2^_Mn_*	0.88	0.91	0.81	0.87	0.91
*S_Mn_*	8349.90	8065.40	10,426.00	3743.00	3048.70
*R^2^_Mw_*	0.79	0.87	0.61	0.97	0.97
*S_Mw_*	13,406.00	11,761.00	17,168.00	1973.80	2118.90

## Data Availability

The data presented in this study are available on request from the corresponding author.

## Supplementary Materials

The following supporting information can be downloaded at: https://www.mdpi.com/article/10.3390/polym14122352/s1, Figure S1: Molecular weight distributions for Exp 2: (a) MWD and the deconvolution 17.7 % of conversion, (b) Evolution of the MWD in conversion.

## Abbreviations

NdV_3_	Neodymium versatate.
DIBAH	Diisobutylaluminum hydrade.
Me_2_SiCl_2_	Dimethyldichlorosilane.
*Np*	Number of polymer chains per neodymium atom.
*M*	β-myrcene monomer.
*C-hex*	Cyclohexane.
[*M*]_0_	β-myrcene monomer concentration at t = 0.
*Nd*	Neodymium catalyst.
[*Nd*]_0_	Neodymium catalyst concentration at t = 0.
*AlR_x_*	Alkyl-aluminum cocatalyst (chain transfer agent during polymerization).
[*AlR_x_*]_0_	Chain transfer agent concentration at t= 0.
*R_H_Cl_2_*	Halide donor.
[*R_H_Cl_2_*]_0_	Halide donor concentration at t= 0.
*M_n_*	Number average molecular weight.
*M_w_*	Weight average molecular weight.
*M_n_^x^*	Number average molecular weight generated by the active centers of type x (x = I or II).
*M_w_^x^*	Weight average molecular weight generated by the active centers of type x (x= I or II).
$Đ\;,\;\frac{M_{w}}{M_{n}}$	Dispersity index.
MWD	Molecular Weight Distribution.
*C ^I*^, C ^II*^*	Active centers of type I and of type II, * denotes highly unstable species.
*P_n_^I^, P_n_^II^*	Type I and Type II propagating polymer chain of length *n*, active chains.
*P_n_ Al ^I^*	Inactive chains for monomer insertion located on aluminum, dormant polymer of type I.
*P_n_ Al ^II^*	Inactive chains for monomer insertion located on aluminum, dormant species of type II.
*D_n_^I^, D_n_^II^*	Type I and Type II inactive species, dead polymer.
*k_app_*	Apparent rate constant of propagation.
*R*	Universal gas constant.
*T*	Absolute temperature.
*A_0_*	Pre-exponential factor.
*E_a_*	Activation energy.
*k_in,I_*	Initiation rate constant for type I species.
*k_in,II_*	Initiation rate constant for type II species.
*k_p,I_*	Propagation rate constant for type I species.
*k_p,II_*	Propagation rate constant for type II species.
*k_trc,I_*	Chain transfer to cocatalyst rate constant for type I species.
*k_trc,II_*	Chain transfer to cocatalyst rate constant for type II species.
*k_da1,I_*	Deactivation rate constant of type I active centers.
*k_da1,II_*	Deactivation rate constant of type II active centers.
*k_da1,I_*	Deactivation rate constant of the type I propagating polymer.
*k_da1,II_*	Deactivation rate constant of the type II propagating polymer.
*k_tr_*	Rate constant for the reversible transfer forward.
*k_tr1_*	Rate constant for the reversible transfer backward.
$\mu_{k}{}^{I}$	*k-th* moment for the active polymer of type I.
$\nu_{0}{}^{I}$	*k-th* moment for the dead polymer of type II.
$\mu_{k}{}^{II}$	*k-th* moment for the active polymer of type II.
$\beta_{k}{}^{II}$	*k-th* moment for the dormant polymer of type II.
$\nu_{0}{}^{II}$	*k-th* moment for the dead polymer of type II.
$Np_{Exp}$	“Experimental” average number of polymer chains produced by single neodymium atom.
$M_{n\;Theo}$	Number average molecular weight obtained if each neodymium atom were to generate a polymer chain.
$M_{n\;Exp}$	Number average molecular weight experimentally determined by GPC.
$M_{n\;Model}$	Number average molecular weight obtained by the model.
EGF	End-Goup Functionality.
EGF_II_	End-Goup Functionality for polymer chains of Type *II.*
*R_j_^2^*	Coefficient of determination.
$SS_{Tot}^{J}$	Sum of squared deviations between predicted values at the time *i* and the average experimental values for a specific characteristic *j.*
*SSE*	Sum of squared errors.
*S*	Standard deviation.
$SS_{Res}^{J}$	Sum of squared deviations between predicted values at the time *i* and the experimental values at the time *i* for a specific characteristic *j.*
$SS_{Tot}^{J}$	Sum of squared deviations between predicted values at the time *i* and the average experimental values for a specific characteristic *j.*
Log_1_*M*	The logarithm of the molecular weight corresponding to the slice retention time (averaged).
dW/d Log M	Normalized distribution of molecular weights, derivative of Weight fraction with respect to logarithm of molar Mass.
