# Supplementary material for: Terpene Coordinative Chain Transfer Polymerization: Understanding the Process through Kinetic Modeling
Source: Polymers (Basel). 2022 Jun 10;14(12):2352. doi: 10.3390/polym14122352 (PMC9228009; doi:10.3390/polym14122352)
Supplement: Supplementary file 1 [file polymers-14-02352-s001.zip › polymers-1726159-supplementary.pdf]

Supporting Information

Terpene Coordinative Chain Transfer Polymerization: Understanding the Process through Kinetic Modeling

Andrés Ubaldo-Alarcón[1], Florentino Soriano-Corral[1], Teresa Córdova, Iván Zapata-González[1]* and Ramón Díaz-de-León[1]*

[1] Centro de Investigación en Química Aplicada, Enrique Reyna Hermosillo, No.140, Col. San José de los Cerritos, Saltillo 25294, México.

* Correspondence: IZG ivan.zapata@ciqa.edu.mx,Tel.: 52 844 4389830 ext. 1254; RDL ramon.diazdeleon@ciqa.edu.mx, Tel.: 52 844 4389830 ext. 1401

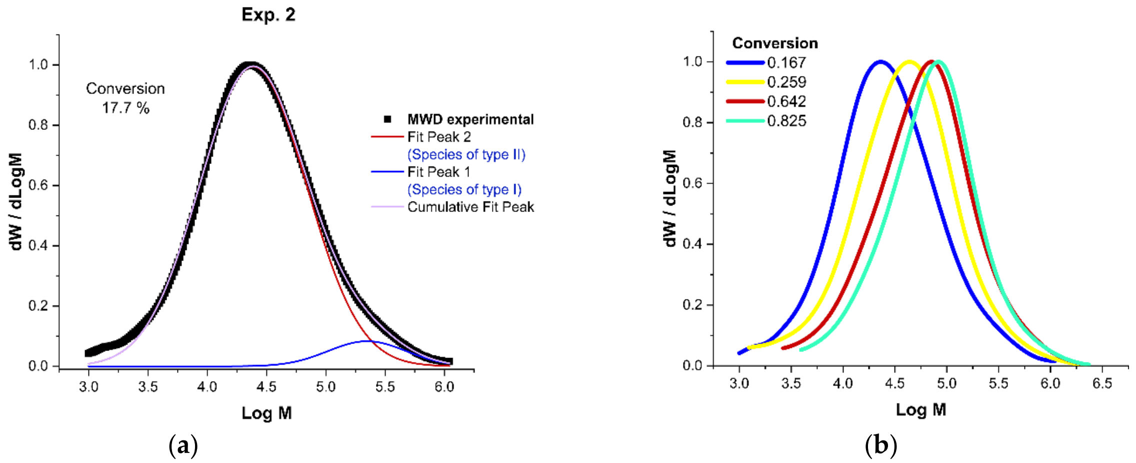

Figure S1. Molecular weight distributions for Exp 2: (**a**) MWD and the deconvolution 17.7% of conversion, (**b**) Evolution of the MWD in conversion.
